# Association between Oxidative Stress and Outcome in Different Subtypes of Acute Ischemic Stroke

**DOI:** 10.1155/2014/256879

**Published:** 2014-05-08

**Authors:** Nai-Wen Tsai, Ya-Ting Chang, Chi-Ren Huang, Yu-Jun Lin, Wei-Che Lin, Ben-Chung Cheng, Chih-Min Su, Yi-Fang Chiang, Shu-Fang Chen, Chih-Cheng Huang, Wen-Neng Chang, Cheng-Hsien Lu

**Affiliations:** ^1^Department of Neurology, Chang Gung Memorial Hospital, Kaohsiung Medical Center, Chang Gung University College of Medicine, 123, Ta Pei Road, Niao Sung District, Kaohsiung, Taiwan; ^2^Department of Biological Science, National Sun Yat-Sen University, Taiwan; ^3^Department of Neurosurgery, Chang Gung Memorial Hospital, Kaohsiung Medical Center, Chang Gung University College of Medicine, 123, Ta Pei Road, Niao Sung District, Kaohsiung, Taiwan; ^4^Department of Radiology, Chang Gung Memorial Hospital, Kaohsiung Medical Center, Chang Gung University College of Medicine, 123, Ta Pei Road, Niao Sung District, Kaohsiung, Taiwan; ^5^Department of Internal Medicine, Chang Gung Memorial Hospital, Kaohsiung Medical Center, Chang Gung University College of Medicine, 123, Ta Pei Road, Niao Sung District, Kaohsiung, Taiwan; ^6^Department of Emergency Medicine, Chang Gung Memorial Hospital, Kaohsiung Medical Center, Chang Gung University College of Medicine, 123, Ta Pei Road, Niao Sung District, Kaohsiung, Taiwan

## Abstract

*Objectives*. This study investigated serum thiobarbituric acid-reactive substances (TBARS) and free thiol levels in different subtypes of acute ischemic stroke (AIS) and evaluated their association with clinical outcomes. * Methods*. This prospective study evaluated 100 AIS patients, including 75 with small-vessel and 25 with large-vessel diseases. Serum oxidative stress (TBARS) and antioxidant (thiol) were determined within 48 hours and days 7 and 30 after stroke. For comparison, 80 age- and sex-matched participants were evaluated as controls. * Results*. Serum TBARS was significantly higher and free thiol was lower in stroke patients than in the controls on days 1 and 7 after AIS. The level of free thiol was significantly lower in the large-vessel disease than in the small-vessel disease on day 7 after stroke. Using the stepwise logistic regression model for potential variables, only stroke subtype, NIHSS score, and serum TBARS level were independently associated with three-month outcome. Higher TBARS and lower thiol levels in the acute phase of stroke were associated with poor outcome. * Conclusions*. Patients with large-vessel disease have higher oxidative stress but lower antioxidant defense compared to those with small-vessel disease after AIS. Serum TBARS level at the acute phase of stroke is a potential predictor for three-month outcome.

## 1. Introduction

Stroke is a major cause of morbidity and mortality worldwide [1]. Inflammation and oxidative stress play important roles in acute ischemic stroke (AIS) [2–4] and the close relationship between inflammation and oxidative stress is now well defined [5, 6]. Acute ischemia leads to increased production of free radicals and reactive oxygen species (ROS) in tissue and plasma through several mechanisms [7], including stimulation of N-methyl-D-aspartate (NMDA) receptors [3], mitochondrial dysfunction [8], activation of neuronal nitric oxide synthase (NOS) [9], and migration of neutrophils and leukocytes that can generate superoxide anions [10]. Although its exact mechanism is not clear yet, oxidative stress is a pivotal event in the setting of ischemic stroke and may contribute to stroke outcome [11, 12].

Oxidative stress has been defined as “an imbalance between oxidants and antioxidants in favor of oxidants, potentially leading to damage” [13]. To assess oxidants, the accumulation of malondialdehyde (MDA), an end-product of peroxidative decomposition of polyenic fatty acids in the lipid peroxidation process, in tissues is indicative of the extent of lipid peroxidation. Measured as thiobarbituric acid-reactive substances (TBARS), MDA is used as an indicator of oxidative damage for several diseases [14].

On the other hand, the antioxidant defense system has been studied in stroke patients as regards enzymes, including superoxide dismutase (SOD) and glutathione peroxidase [15, 16], and nonenzymatic antioxidants like retinol, ascorbic acid, *α*-tocopherol, carotenoids, and uric acid [3, 17, 18]. Most of these studies are cross-sectional or have short follow-up periods after stroke.

Under the hypothesis that the level of oxidative stress is increased and may be diverse in different subtypes of stroke, this study evaluated longitudinal changes in serum oxidant and antioxidant levels after ischemic stroke to determine their value in predicting short-term outcome. Serial changes of serum TBARS and free thiol were measured in different subtypes during the first month after stroke and the possibility of using these markers for predicting three-month outcome was assessed.

## 2. Patients and Methods

### 2.1. Subjects and Design

From August 2010 to July 2012, consecutive patients with AIS who were admitted to the Neurology Department of Chang Gung Memorial Hospital-Kaohsiung were evaluated. Acute ischemic stroke was defined as an acute onset loss of focal cerebral function persisting for at least 24 hours. Diagnosis was based on clinical presentation, neurologic examination, and results of brain magnetic resonance imaging (MRI) with diffusion-weighted imaging (DWI). Patients aged 18–80 years with acute noncardioembolic ischemic stroke were included and divided into two major etiologic subtypes (i.e., large-artery atherosclerosis and small-artery occlusion) according to the TOAST (Trial of Org 10172 in Acute Stroke Treatment) classification [19]. For comparison, 80 age- and sex-matched subjects with no clinical evidence of acute cerebral infarction within one year were enrolled as the control group. The hospital's Institutional Review Committee on Human Research approved the study protocol and all participants provided informed consent.

Patients with cardioembolic stroke, other determined causes and undetermined causes of stroke, and those with underlying neoplasm, end-stage renal disease, liver cirrhosis, and congestive heart failure were excluded. Clinical examination, electrocardiography, and cardiac ultrasound were conducted to exclude cardioembolic stroke. Patients with fever or any infectious disorder within the first week after acute stroke were also excluded.

### 2.2. Clinical Assessments and Treatment

All of the participants underwent complete neurologic examination. Brain MRI with DWI, extracranial carotid sonography, and transcranial color-coded sonography were performed during the hospitalization. The therapeutic regimens for AIS were based on the American Heart Association (AHA)/American Stroke Association (ASA) guidelines [20]. Neurologic deficits due to stroke were assessed using the National Institutes of Health Stroke Scale (NIHSS). The therapeutic outcomes were evaluated by the modified Rankin Scale (mRS) at three months after stroke. Good outcome was defined as a three-month mRS of 0–2 without any cardiovascular event. Poor outcome was defined as mRS of 3–6 [21].

### 2.3. Determination of Serum Malondialdehyde Content

Blood samples were collected by venipuncture of forearm veins from patients within 48 hours of the stroke (presented as day 1) and on days 7 and 30 after stroke. Serum MDA was measured using the TBARS assay. The concentration of TBARS was assessed based on the method of Huang et al. [22]. TBARS reagent (1 mL) was added to a 0.5 mL aliquot of serum and heated for 20 minutes at 100°C. The antioxidant, butylated hydroxytoluene, was added before heating the samples. After cooling on ice, the samples were centrifuged at 840 g for 15 min. Absorbance of the supernatant was read at 532 nm. Blanks for each sample were prepared and assessed in the same way to correct for the contribution of A532 to the sample. The TBARS results were expressed as MDA equivalents using 1,1,3,3-tetraethoxypropane.

### 2.4. Assessment of Serum Free Thiol Content

The ability of antioxidative defense in response to increased oxidative damage was evaluated by measuring the serum level of total reduced thiols because thiols were physiologic free radical scavengers. Serum free thiols were determined by directly reacting thiols with 5,5-dithiobis 2-nitrobenzoic acid (DTNB) to form 5-thio-2-nitrobenzoic acid (TNB). The amount of thiols in the sample was calculated from absorbance, as determined using the extinction coefficient of TNB (A412 = 13,600 M^−1^ cm^−1^).

### 2.5. Statistical Analysis

Data were presented as mean ± SEM. Continuous variables, including age, cell count, lipid profile, hemoglobin A1c (HbA1c), blood pressure, and serum free thiol and TBARS, were analyzed by independent* t*-test among groups. Chi-square test or Fisher's exact test was used to compare proportions among groups. Repeated measures of ANOVA were used to compare serum free thiol and TBARS at different time points (within 48 hours and on days 7 and 30 after stroke).

Scheffe's multiple comparison was used to analyze the intraindividual courses of parameters over time. These were then compared among patients with small-vessel and large-vessel diseases. Multiple logistic regression analyses determined the independent influence of different predictive variables on clinical outcome. Statistical significance was set at *P* < 0.05. All statistical calculations were performed using the SAS software package, version 9.1 (2002, SAS Statistical Institute, Cary, NC, USA).

## 3. Results

### 3.1. Baseline Characteristics of Patients with Small-Vessel and Large-Vessel Diseases

Of the 120 patients with acute noncardioembolic ischemic stroke, 20 were excluded for various infections or fever in the first week after acute stroke (*n* = 6), cardioembolic stroke (*n* = 5), end-stage renal disease (*n* = 5), and gastrointestinal bleeding in the acute stage (*n* = 4). The remaining 100 patients included 75 with small-vessel occlusion and 25 with large-vessel atherosclerosis. Based on their baseline characteristics and laboratory data (Table 1), there were no significant differences in vascular risk factors and in white blood cell (WBC), red blood cell (RBC) count, platelet count, and serum levels of total cholesterol, LDL-cholesterol, triglyceride, and HbA1c. Serum concentration of free thiol and TBARS were also not different between the two groups.

### 3.2. Changes in Serum TBARS and Free Thiol among Patients with Small-Vessel and Large-Vessel Diseases

**Figure 1:**
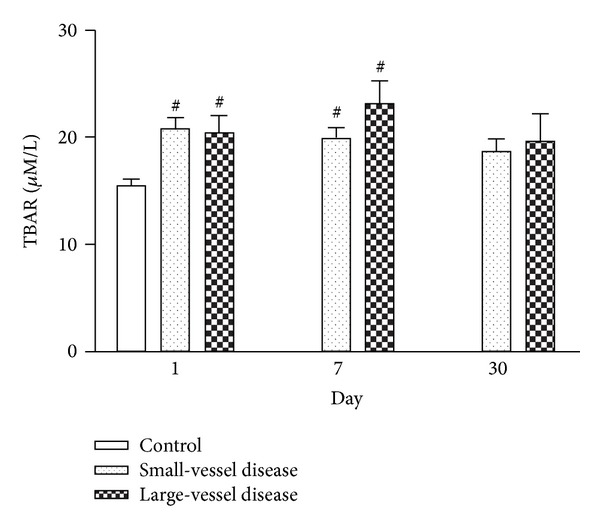
Serial changes in serum TBARS among patients with small-vessel and large-vessel diseases and in the controls at various time points after stroke. ^#^ *P* < 0.05 compared to controls.

Serial changes in serum concentration of TBARS among patients groups and in the controls (Figure 1) revealed that the concentration of TBARS was significantly higher in stroke patients than in the controls on days 1 and 7 after AIS (*P* < 0.05). At the three different time points, the levels of TBARS were similar between patients with large-vessel disease and those with small-vessel disease.

**Figure 2:**
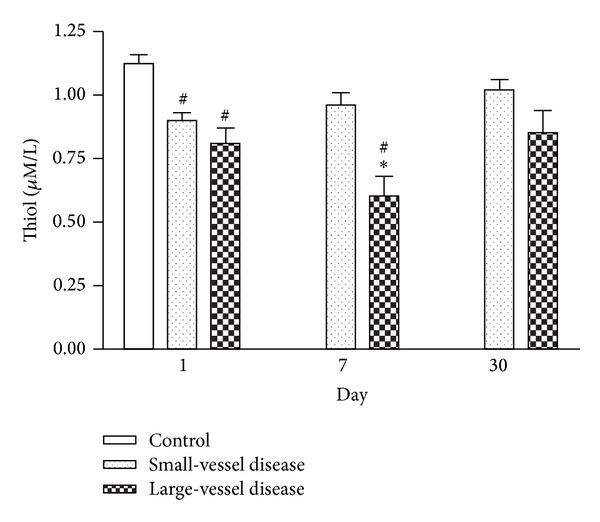
Serial changes in serum free thiol among patients with small-vessel and large-vessel diseases and in the controls at various time points after stroke. *P < 0.05 compared to the small vessel group; ^#^ *P* < 0.05 compared to the controls.

Serial changes in serum concentration of free thiol among patients with small-vessel and large-vessel diseases and in the controls (Figure 2) demonstrated that the concentration of free thiol was significantly lower in stroke patients than in the controls on day 1 after AIS (*P* < 0.05). Free thiol concentration was also significantly lower in the large-vessel disease group than in the small-vessel disease group on day 7 after stroke (*P* < 0.05). Thereafter, the level of free thiol gradually increased until it became similar to that of the controls on day 30 after stroke. Repeated ANOVA with Scheffe's multiple comparison showed significantly different free thiol levels between patients with small-vessel disease and those with large-vessel disease at three different time points (within 48 h and on days 7 and 30 after stroke) (*P* < 0.05).

### 3.3. Factors Predictive of Clinical Outcome

Potential prognostic factors of the 100 stroke patients were listed in Table 2. No one died during the three-month followup, and 80 patients had good outcomes while 20 had poor outcomes. Statistical analysis revealed that stroke subtype, NIHSS score, and serum free thiol and TBARS levels on days 1 and 7 after stroke were significantly different between the good and poor outcome groups.

Potential variables such as age, sex, stroke subtype, blood pressure, HbA1C, total cholesterol, HDL, LDL, and TBARS and free thiol levels on admission were analyzed using a stepwise logistic regression model. Only the stroke subtype (OR: 0.014, 95% CI: 0.001–0.325; *P* = 0.008), NIHSS score (OR: 1.55, 95% CI: 1.11–2.16; *P* = 0.01), and serum TBARS on day 7 after stroke (OR: 1.37, 95% CI: 1.14–1.65; *P* = 0.001) were independently associated with three-month outcome. Any increase in TBARS concentration by one *μ*M/L worsens the poor outcome rate by 37%.

### 3.4. Serial Changes in Serum TBARS and Free Thiol between Groups with Good and Poor Outcomes

**Figure 3:**
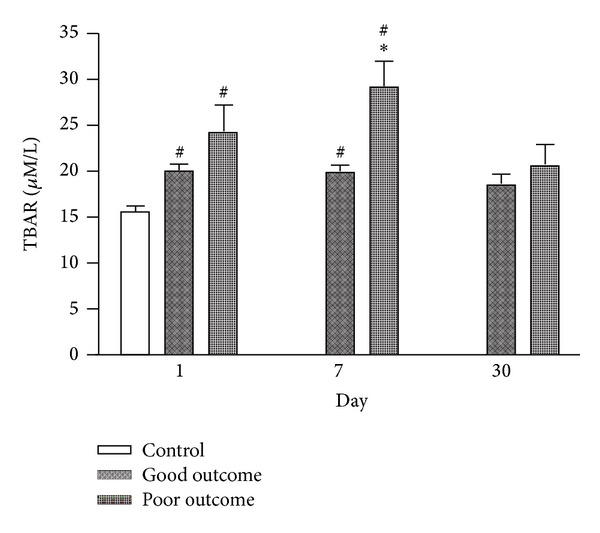
Serial changes in serum TBARS among patients with good and poor outcomes and in the controls at various time points after stroke. *P < 0.05 compared to the small vessel group; ^#^ *P* < 0.05 compared to the controls.

Changes in serum TBARS between groups with good and poor outcomes (Figure 3) revealed a gross increase in TBARS level in the poor outcome group during the acute stage after stroke. The concentration of TBARS was significantly higher in the poor outcome group than in the good outcome group on day 7 after stroke (*P* < 0.05). These levels gradually decreased thereafter and no significant difference existed between the two groups on day 30 after stroke. Repeated ANOVA with Scheffe's multiple comparison revealed significantly different serum TBARS levels on three different time points between the two groups (*P* < 0.05).

**Figure 4:**
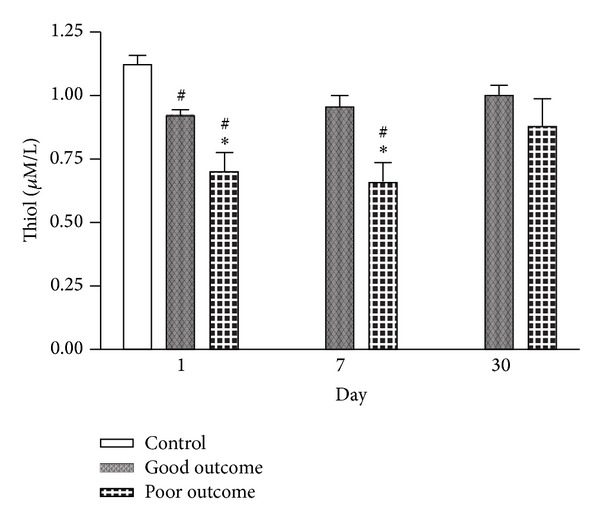
Serial changes in serum free thiol among patients with good and poor outcomes and in the controls at various time points after stroke. *P < 0.05 compared to the small vessel group; ^#^ *P* < 0.05 compared to the controls.

Changes in serum concentration of free thiol among patients with good and poor outcomes, and in the controls (Figure 4) revealed that the concentration of free thiol was significantly lower in the poor outcome group than in the good outcome group on days 1 and 7 after stroke (*P* < 0.05). Repeated ANOVA with Scheffe's multiple comparison showed significantly different free thiol levels between the good and poor outcome groups at three different time points (within 48 h and on days 7 and 30 after stroke) (*P* < 0.05).

## 4. Discussion

The present study has four major findings. First, patients with AIS in the acute phase had significantly higher TBARS and lower free thiol levels than the controls. Second, the level of free thiol is significantly lower in patients with large-vessel disease than in those with small-vessel disease on day 7 after stroke. Third, the higher TBARS and lower free thiol levels in the acute phase of AIS is associated with poor outcome. Lastly, the most important finding in this study is that TBARS level on day 7 after stroke is an independent predictive factor of three-month outcome.

The results on the MDA level changes over time in stroke patients are controversial. Some researchers observed higher erythrocyte MDA levels in the very early phase of stroke, with subsequent decline in the levels of the controls [23], while others report an MDA increase only in some days after acute stroke [24]. Gariballa et al. found that TBARs levels were constantly higher in AIS patients compared to those of the controls [15]. Consistent with a previous study [25], the longitudinal observation here shows higher levels of TBARS in stroke patients than in controls upon hospital admission, and these levels persist in the next 7 days. Thereafter, TBARS levels gradually decrease to the level as controls a month after the stroke. The discrepancy in results may be due to methodological factors, including sample handling, storage, and preparation prior to the performance of the biomarker assay. Moreover, stroke is a pathologically heterogeneous disease and baseline risk factors may be etiologically different.

Furthermore, the current study demonstrates that antioxidant levels, measured by free thiol, are much lower in patients with large-vessel cerebral infarction than in those with small-vessel infarction on day 7 after stroke. The level of free thiol gradually increases until the difference is no longer significant one month after the stroke. These suggest that oxidant/antioxidant balance is related to the different pathogenesis in the two major subtypes of noncardioembolic stroke. The pathogenesis of small-vessel infarction is lipohyalinosis [26], while atherothrombosis is the major cause of large-vessel cerebral infarction [27]. Thus, different subtypes of ischemic stroke have different pathogenesis, with consequences on oxidative stress.

Oxidative stress is an important contributor to the pathophysiologic sequelae of stroke. A correlation of MDA level with infarct size, clinical stroke severity, and patient outcome has been observed [15, 25]. Since plasma level of oxidized LDL (Ox-LDL) is thought to reflect the oxidative status of the whole body, a previous study has shown that higher Ox-LDL level in the acute phase of AIS is an independent predictor of poor outcome three months after stroke [28]. The results here reveal that the level of TBARS on day 7 but not day 1 after stroke is much higher in patients with poor outcome than in those with good outcome (Figure 3). Moreover, TBARS level on day 7 after stroke is an independent predictive factor of three-month outcome. These findings suggest that oxidative stress is progressive after stroke and contributes to further neurologic damage in particular cases.

On the other hand, antioxidant vitamin concentrations are associated with neurologic damage and stroke prognosis [29, 30]. The antioxidant defense system, measured by SOD activity, is inversely correlated with infarct size and the severity of neurologic damage [15, 16]. The data here also confirms that the lower antioxidant (free thiol) level in the acute phase of stroke is associated with poor outcome.

Although other inflammatory biomarkers like high-sensitivity C-reactive protein (hs-CRP) and leukocytes have been reported to be useful in predicting clinical outcome after stroke [31, 32], there has been no head-to-head study to date that compares these markers in terms of predictive value. A previous research demonstrates that statin therapy reduces serum hs-CRP level and oxidized LDL in patients after AIS [33]. A prospective study is warranted to evaluate the predictive value of these biomarkers on stroke outcome.

Some limitations of this study should be acknowledged. First, the measurement of only few biomarkers of oxidative damage cannot be considered a valid tool for exploring a multifaceted, complex oxidant/antioxidant imbalance after acute stroke. Second, the oxidant/antioxidant balance of stroke patients may be influenced by a multitude of parameters, including age, sex, smoking habit, alcohol consumption, physical activity, and vitamin supplementation. Third, oxidative stress may be influenced by other drugs (e.g., antiplatelet, angiotensin II type 1 receptor blockers, and antidiabetics). Since the use of these drugs depends on the preference of the attending physician, this may cause potential bias in statistical analysis and in drawing conclusions. Nonetheless, the sample size is not large and the number of variables considered in the stepwise logistic regression analysis is small. Hence, the maximum likelihood estimates of the coefficients are valid in the analysis.

In conclusion, patients with AIS have significantly higher TBARS and lower free thiol levels than healthy controls. The level of free thiol is significantly lower in patients with large-vessel disease than in those with small-vessel disease in the acute phase of stroke. Serum TBARS on day 7 after stroke is an independent predictive biomarker of three-month stroke outcome.

## Figures and Tables

**Table 1:** Baseline characteristics and laboratory data between small-vessel and large-vessel disease.

	Small vessel disease	Large vessel disease	*P* value
	(*n* = 75)	(*n* = 25)	
Age (y) (mean ± SD)	61.1 ± 11.5	64.6 ± 8.8	0.17
Sex (male) (*n*)	60	17	0.27
Hypertension (*n*)	56	22	0.26
Diabetes mellitus (*n*)	32	10	0.82
Dyslipidemia (*n*)	37	12	0.91
Coronary artery disease (*n*)	5	2	0.82
White blood cells (×10^3^/mL)	7.4 ± 0.3	8.1 ± 0.5	0.22
Red blood cells (×10^6^/mL)	4.8 ± 0.1	4.5 ± 0.1	0.11
Platelet counts (×10^4^/mL)	21.1 ± 0.7	19.8 ± 1.2	0.30
Total cholesterol (mg/dL)	181.2 ± 3.8	194.6 ± 11.6	0.15
LDL-cholesterol (mg/dL)	109.9 ± 3.7	123.4 ± 9.9	0.12
Triglyceride (mg/dL)	134.7 ± 7.7	134.9 ± 13.3	0.99
HbA1c (%)	7.0 ± 0.2	6.4 ± 0.3	0.15
Free thiol (*μ*M/L)	0.90 ± 0.03	0.81 ± 0.06	0.19
TBARS (*μ*M/L)	19.7 ± 1.2	20.7 ± 2.6	0.85

**Table 2:** Prognostic factors in patients with acute ischemic stroke.

	Good outcome (*n* = 80)	Poor outcome (*n* = 20)	Crude OR (95% CI)	*P* value	Adjusted OR (95% CI)	*P* value
Age (year)	61.2 ± 11.5	65.1 ± 8.0	1.04 (0.99–1.09)	0.16	—	—
Sex (male) (*n*)	62	15	0.87 (0.28–2.72)	0.81	—	—
Hypertension (*n*)	61	17	1.77 (0.47–6.68)	0.55	—	—
Diabetes mellitus (*n*)	32	10	1.50 (0.56–4.01)	0.46	—	—
Hyperlipidemia (*n*)	38	11	1.35 (0.51–3.61)	0.62	—	—
Cardiac disease (*n*)	5	2	1.67 (0.30–9.30)	0.80	—	—
NIHSS score on admission			1.35 (1.16–1.58)	<0.001	1.55 (1.11–2.16)	0.01
Stroke subtype (large/small)			21.0 (6.26–70.4)	<0.001	0.01 (0.001–0.33)	0.008
Small vessel disease	70	5			—	—
Large vessel disease	10	15			—	—
With statin therapy	35	11	1.49 (0.56–4.00)	0.46	—	—
Laboratory data on admission					—	—
White blood cells (×10^3^/mL)	7.4 ± 0.2	8.3 ± 0.6	1.17 (0.96–1.43)	0.12	—	—
Hemoglobin (g/dL)	13.8 ± 0.2	13.6 ± 0.3	0.91 (0.69–1.22)	0.53		
Red blood cells (×10^6^/mL)	4.8 ± 0.1	4.5 ± 0.1	0.50 (0.24–1.07)	0.07	—	—
Platelet counts (×10^4^/mL)	21.2 ± 0.6	18.9 ± 1.4	0.99 (0.98–1.00)	0.12	—	—
Total cholesterol (mg/dL)	185.7 ± 4.6	180.0 ± 8.6	0.99 (0.98–1.01)	0.58	—	—
LDL-cholesterol (mg/dL)	114.2 ± 4.2	109.6 ± 7.7	0.99 (0.98–1.01)	0.62	—	—
HDL-cholesterol (mg/dL)	43.8 ± 1.0	45.6 ± 3.0	1.02 (0.97–1.07)	0.48	—	—
Triglyceride (mg/dL)	136.3 ± 7.6	128.3 ± 13.8	0.99 (0.99–1.01)	0.63	—	—
HbA1c (%)	6.8 ± 0.2	7.3 ± 0.6	1.14 (0.90–1.45)	0.28	—	—
Systolic BP (mmHg)	147.1 ± 2.8	140.1 ± 4.4	0.99 (0.97–1.01)	0.28	—	—
Diastolic BP (mmHg)	84.5 ± 1.5	81.1 ± 2.8	0.98 (0.94–1.02)	0.31	—	—
Free thiol on admission (*μ*M/L)	0.92 ± 0.03	0.70 ± 0.07	0.04 (0.01–0.32)	0.002	—	—
TBARS on admission (*μ*M/L)	19.9 ± 0.72	24.1 ± 3.0	1.05 (0.99–1.11)	0.06	—	—
Free thiol on day 7 (*μ*M/L)	0.96 ± 0.04	0.66 ± 0.08	0.12 (0.03–0.52)	0.005	—	—
TBARS on day 7 (*μ*M/L)	18.4 ± 0.77	29.1 ± 2.8	1.14 (1.07–1.22)	<0.001	1.37 (1.14–1.65)	0.001
Free thiol on day 30 (*μ*M/L)	1.00 ± 0.04	0.88 ± 0.11	0.29 (0.05–1.75)	0.78	—	—
TBARS on day 30 (*μ*M/L)	19.7 ± 1.24	20.4 ± 2.4	1.01 (0.95–1.07)	0.82	—	—

Abbreviations: BP: blood pressure; HbA1c: hemoglobin A1c.
